# Adherence Patterns of Patients Using Remote Patient Management After Myocardial Infarction: Mixed Methods Persona Approach

**DOI:** 10.2196/56236

**Published:** 2025-08-18

**Authors:** Sara M Hondmann, Laura Schrauwen, Thomas Reijnders, Esmee Stoop, Andrea WM Evers, Valentijn T Visch, Douwe E Atsma, Veronica R Janssen

**Affiliations:** 1 Health, Medical and Neuropsychology Unit, Institute of Psychology, Leiden University, Wassenaarseweg 52, Leiden, 2333 AK, The Netherlands, 31 71 527 27 27; 2 Department of Industrial Design Engineering, Delft University of Technology, Delft, The Netherlands; 3 Department of Information Technology and Digital Innovation, Leiden University Medical Center, Leiden, The Netherlands; 4 Medical Delta, Leiden University, Delft University of Technology, Erasmus University, Delft, The Netherlands; 5 Department of Cardiology, Leiden University Medical Center, Leiden, The Netherlands

**Keywords:** cardiovascular disease, eHealth, personas, remote patient management, adherence, blood pressure

## Abstract

**Background:**

Remote patient management (RPM) using smartphone-enabled health monitoring devices (SHMDs) can be an effective, value-added part of cardiovascular care. However, cardiac patients’ adherence to RPM is variable. Personas are fictional representations of users with common behaviors, needs, and motivation and can thereby help guide tailoring of interventions to be meaningful and possibly more effective. Personas can be used to understand the needs of the patient group and guide tailoring toward more personalized and effective eHealth intervention.

**Objective:**

The aim of this study was to develop data-driven personas for patients with myocardial infarction (MI) based on both quantitative and qualitative results.

**Methods:**

This study used a mixed methods design involving (1) database analysis of patients with MI (N=261) SHMD usage data (blood pressure [BP], weight, step count) over the course of a one-year care track and (2) semistructured interviews with patients with MI (N=16) currently using SHMDs. Overall, 12-month adherence rates were calculated based on the number of weeks patients performed the prescribed home measurements with the SHMDs.

**Results:**

A cluster analysis was conducted on the self-monitoring data resulting in four distinctive usage patterns labeled as stiff starting (low adherent in first 6 weeks: 13%, 34/261 of users), temporary persisting (decreasing adherence: 24%, 62/261), loyally persisting (continuously adherent: 26%, 68/261), and negligent quitting (nonadherent: 37%, 97/261). Health outcomes (BP, step count, and weight) were analyzed based on these patterns. More adherent usage patterns show better controlled BP when compared to less adherent usage patterns, suggesting that adherence is associated with health outcomes. Patient experiences regarding adherence or nonadherence to the RPM relating to the four distinctive usage patterns were uncovered by means of semistructured interviews, providing insight into adherence factors most relevant for each of the clusters. Thus, 4 distinct personas were developed by data collection (database analysis and semistructured interviews), persona segmentation, and persona creation, named Tamara, Sam, Peter, and Kim.

**Conclusions:**

This study identified 4 personas regarding adherence experiences and usage patterns of patients within an RPM care track. Adherent usage patterns were characterized by improved BP and step count. These personas can guide future tailoring of eHealth interventions to maximize patient adherence.

## Introduction

Cardiovascular disease (CVD) is a leading cause of death and disability worldwide [1]. CVD can partially originate and be perpetuated by several modifiable lifestyle risk factors including an unhealthy diet, insufficient physical activity, and smoking leading to elevated body weight and blood pressure (BP) [2-4]. Addressing these modifiable risk factors can improve symptoms, health outcomes, and overall quality of life [5]. Various patient groups, for example, those who have experienced an ST-elevation myocardial infarction (STEMI) or non-ST acute coronary syndrome (NST-ACS), benefit from tight control of their BP, weight, and physical activity [6]. A promising method to facilitate rehabilitation and tackle lifestyle risk factors is the use of remote patient management (RPM), adjunct to clinic care [7]. RPM is an intervention type that makes use of eHealth methods to deliver care. eHealth comprises a variety of methods to deliver care and promote health using applications, websites, digital health records [8], and smartphone-enabled health monitoring devices (SHMDs). Various studies show that eHealth is as, or more, effective than standard care [9-11]. Close monitoring through RPM can help to reduce hospital admissions, disease progression, risk factors, and allow for early intervention [12,13].

“The Myocardial Infarction (MI) Box” is an RPM intervention for cardiology patients after myocardial infarction in the home context. It makes use of SHMDs to monitor and provide care to patients discharged from the Leiden University Medical Center (LUMC) after a STEMI or NST-ACS event [6,11] Data from the SHMDs are automatically transferred to and integrated into the electronic medical record (EMR) [11], which provides clinicians with more accurate data to make treatment decisions and give patients feedback on important health parameters, such as BP, body weight, physical activity, and heart rhythm for 1 year after discharge [6]. Furthermore, the MI Box is a tool for the stimulation of a healthy lifestyle. Treskes et al [11] demonstrated through a noninferiority trial that The MI Box as stand-alone aftercare treatment is as effective as standard cardiovascular care. However, like other eHealth interventions, it is curtailed by variable adherence. This nonadherence can lead to a loss of guidance for these patients, a worsening of symptoms, and at worst another cardiac event [7,12].

Adherence to the technology within eHealth is variable, including users who adhere long term, those who stop usage after a short period, those who do not use the functionalities as intended, and those who do not use the devices at all [14-16]. Reasons for this spectrum of adherence could be that needs, preferences, and capacities of users are not sufficiently considered [17], leading to a misalignment between the intervention and the users’ needs and abilities. Factors influencing (non)adherence in cardiac interventions are varying, including intrapersonal, clinical, health system, and logistical factors [18]. The duration of the intervention possibly influences adherence, with longer interventions having adverse effects on adherence [19]. Tailoring based on theoretical, behavioral, and demographic variables has been associated with more effective interventions [20]. The literature highlights the importance of balancing personalized and generalizable approaches to improve outcomes. In this study, this balance is achieved using data-driven personas, while in other studies (eg, ref iris and foot), this is accomplished through biometric identification systems, which consider both unique individual characteristics and broader applicability to diverse populations [21]. Personas are a method to determine tailoring strategies to correct this misalignment and enhance adherence [22].

Personas are representations of users with common characteristics, behaviors, and needs [23,24] and can be constituted of quantitative and qualitative data [25,26]. Personas can help prioritize problems, direct focus on specific characteristics and needs of subpopulations, highlight and challenge assumptions about populations, and sensitize those creating interventions on how they differ from the patients they serve. Personas are narratives that highlight the user perspective and can thereby guide conversations and changes to the interventions made by health care professionals and designers [22,23,25,27]. For example, personas can guide cocreative sessions by providing a narrative with which stakeholders can discuss issues facing the users and brainstorm design solutions. Research in patients with cancer, diabetes, and heart failure has used personas to gain a better understanding of the population, which allowed for distinguishing behavioral factors influencing acceptance, distinguishing different subpopulations’ needs, and influencing implementation [25,28,29]. Although there is not one definitive way to create personas since they are unique to each population and context, there are three general steps in their creation: data collection, persona segmentation, and persona creation [22]. Data collection comprises a combination of both qualitative and quantitative methods, either separately or together. Persona segmentation is creating groups based on similarities in demographic or behavioral variables. Finally, the persona creation step involves the designing of the layout of the persona and what information is included [22]. To our knowledge, personas based on qualitative and quantitative data representing patients using RMP or SHMDs have not yet been published. Personas based on mixed methods can be valuable for they describe the biopsychosocial complexities of adherence [30], thereby providing insight into tailoring methods to improve compliance and ensuring that interventions are patient-centered.

Therefore, the aim of this study was to develop personas of patients using SHMDs in RPM care. We used a mixed methods approach separated into three steps: data collection, persona segmentation, and persona creation. The data collection is further divided into 2 steps: a database analysis of self-management data and generative semistructured interviews. The developed personas can be used for tailoring of RPM interventions, which could lead to increased adherence and improved user experience. Furthermore, this mixed method approach could be generalized to other eHealth interventions striving to understand their target population and enhance adherence.

## Methods

### Materials: The MI Box

The MI Box is an RPM intervention including four SHMDs: a BP monitor (Wireless Blood Pressure Monitor; Withings), a step counter (Pulse Ox; Withings), a weight scale (Smart Body Scale Analyzer; Withings), and a single-lead electrocariography (ECG) device (Kardia; AliveCor Inc). The devices communicated with the device-dedicated app on the smartphone via Bluetooth [11]. Patients were followed for one year. This patient population has the same diagnosis and all patients have followed a guideline-driven medical therapy (GDMT). This protocol, standardized with regard to interventions, evaluations, and medication, helps control for confounding variables related to treatment variability, isolating the effects of the RPM intervention and its adherence. Patients can immediately see their measurements, and the data is automatically sent to the LUMC and included in the patient dossier [11]. The data are evaluated multiple times per week by the clinician to monitor the health status of each patient. Thus, the therapeutic regimen could be revised based on the results of measurements as well as symptoms. Furthermore, patients consulted with their physician or nurse practitioner 4 times (1, 3, 6, and 12 months after discharge) to discuss rehabilitation, medication, and any other matters of concern. The first and third meetings were video consults, and the second and fourth were outpatient clinic visits. Patients were prescribed to take at least 1 measurement with each device per week.

### Data Collection

The data collection comprised 2 steps. First, self-management data from Box users who have completed the year-long care track was clustered by type and frequency of the measurements taken (BP, steps, and weight). These variables were selected to differentiate the clusters based purely on usage and thereby provide information on adherence of users. Furthermore, type and frequency of measurements were consistently available in the EMR and reliable indicators of use. Health outcomes (BP, weight, and step count) were analyzed based on these clusters. Second, generative interviews were conducted with patients currently using the SHMDs to understand their experience regarding (non)adherence and enrich the found patterns. This integrated analysis enables the creation of personas.

### Database Analysis

The dataset for this analysis was collected from The MI Box database, a module in the EMR where all patients’ measurements were continuously stored in real-time. Data were acquired from May 2017 till January 2020 and included data from patients at least one year after discharge, allowing us to find usage patterns on a yearly basis. The obtained longitudinal dataset included a pseudo-ID for each subject, age, gender, the measurement type (BP, weight, and steps), measurement value, and the corresponding timestamp of each measurement. ECG measurements were not available for analysis since these were not saved in the EMR. The dataset was thereby obtained with anonymized data safeguarding participants’ privacy.

### Variables

Prior to the analysis, invalid measurements were removed: a low cut-off point of ≥100 steps per day was applied to the pedometer measurements, as this indicated that the device was not worn but only moved around [31]. Subsequently, all measurement data were aggregated from days to frequencies per week within a range of 0‐54 weeks (duration of the care track with a margin of 2 weeks). Then, the variables were transformed according to the generic minimal instructions communicated by the hospital: using each device at least once a week. Finally, these device-specific variables were summed, resulting in one time series array per subject consisting of 54 variables with values ranging from 0‐3. A zero indicated that no devices were used, and a three indicated that all three different devices were used in a specific week.

### Cluster Analysis

In order to identify distinct characteristics in heterogeneous samples and cluster them into homogeneous and meaningful groups, a cluster analysis was conducted based on users’ usage pattern over time [27,32]. The cluster analysis was based on each user’s use of The MI Box over the 1-year care track and not on demographic variables, such as age and sex, or on the clinical measures (CMs). In other words, the resulting clusters were purely based on similarity of usage patterns. A k-means clustering algorithm including the dynamic time warping (DTW) distance measure was used to determine the different clusters. DTW is a distance measure for dynamically comparing time series data when the time indices between comparison data points do not synchronize perfectly [33]. The cluster algorithm was run for k ranging between 2‐8. To determine the optimal number of clusters, average silhouette scores for the different values of k were calculated. Values approaching 1 indicated that the data point was in the correct cluster, and values approaching -1 indicated that the data point was in the wrong cluster [34]. These clusters were then named and defined into user patterns.

### Usage Pattern Comparisons

Two approaches to compare the usage patterns were conducted. First, the demographic variables per cluster were explored. Second, an explorative ANOVA was performed to examine differences between the clusters regarding their health outcomes (mean Systolic BP [SBP], diastolic BP [DBP], weight, and steps in month 1, 6, and 12). This second analysis was performed with an objective to compare mean values between clusters, which can be of direct clinical relevance. Multiple univariate ANOVAs were used for normally distributed variables, and the nonparametric Kruskal-Wallis test for nonnormally distributed variables. Furthermore, to compare the health outcomes over time within each cluster, multiple repeated measures ANOVAs were executed for the health outcomes in month 1, month 6, and month 12. All analyses were judged at the threshold *P*<.05. To correct for multiple testing, a Bonferroni correction was used.

### Software

The cluster analysis and corresponding data visualization tasks were carried out using Python 3.8 and the following libraries: NumPy for numerical computations [35], Pandas for data structures [36], Plotly for data visualizations [37], Scikit-learn for clustering [33], Tslearn for DTW clustering [38], and Streamlit for data visualizations [39]. Statistical analyses for cluster characterization and exploration were performed in the software package IBM SPSS Statistics 26.

### Generative Interviews

#### Participants

A quota sampling strategy for recruiting patients using The MI Box, in collaboration with specialized nurses and cardiologists, was used. Sampling based on usage patterns allowed recruiting a varying group of participants. A decision tree was created to recruit patients based on their usage data. LS and TR both classified participants based on this tree and results were compared until a consensus was reached (refer to Multimedia Appendix 1). Potential participants were approached by the nurse practitioner in the outpatient clinic, and those who expressed interest were contacted by the research team. Once the participants consented, they were formally enrolled and participated in the interviews. To be able to connect a participant to a user profile, participants were required to be in follow-up for at least 6 months. Data of their SHMDs was provided in the EMR.

#### Procedure

One week before the interview, each participant received a paper-based sensitizing booklet with four exercises to complete. Sensitizing booklets are part of the context mapping research method [40] and help prepare participants for an interview. Sensitization allows for a greater and high-quality contribution of the participants, as participants will gain insight into their experiences, enabling them to share this during their interview [40]. The sensitizing booklet included questions about their experience with The Box, positive and negative aspects about the use of The Box, experience with the individual devices, possible changes in lifestyle, and possibility to improve The Box. These questions and answers were discussed further in the semistructured interviews. Furthermore, during the interview, participants were asked to reflect on their usage and the found usage patterns. Semistructured interviews were conducted via videocalling with selected patients based on their personal usage pattern. Interviewees provided written informed consent. The interviews lasted between 30‐60 minutes and were audio-recorded, transcribed, and thematically analyzed using Atlas.ti (by LS and TR) [41,42]. Transcripts were independently coded and then grouped into broader themes, and discrepancies were discussed and resolved.

#### Persona Development

The personas were developed based on the clusters stratified and emergent themes from the quantitative and qualitative methods. This integrates both data-driven and qualitative-focused personas development methods and includes 3 steps as described by Alsaadi and Alahmadi (2021): data collection, persona segmentation, and persona creation [22].

### Ethical Considerations

The Medical Research Ethics Committee Leiden The Hague Delft waived ethical approval for this study as the Dutch law concerning research involving human beings (Dutch abbreviation WMO) did not apply to this protocol (protocol N21.048).

## Results

### Database Analysis

#### Demographics

In total, 263 subjects were available to be included; however, 2 subjects were excluded as outliers due to one being a test subject and the other having unrealistic measurement frequency. In total, 261 subjects were included in the analyses. The average age was 58 (SD 10.59) years and 77.4% (202/261) were male. Overall, 29% (76/261) of the participants sent at least one measurement each week for 52 weeks and 53% (138/261) of the patients sent data for more than 80% (41.6/52) of the weeks within the care track.

#### Usage Patterns

**Figure 1.**
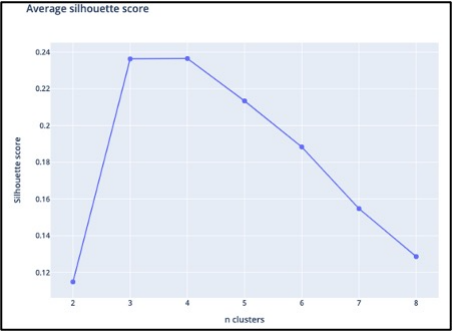
Average silhouette scores of K ranging between 2 and 8.

**Figure 2.**
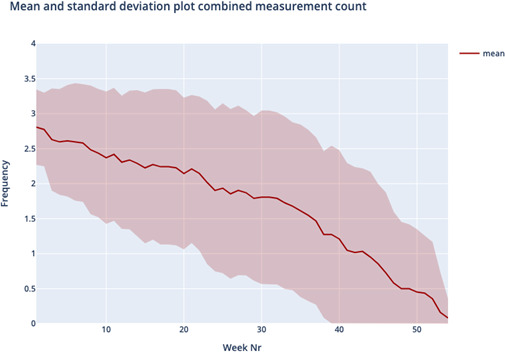
Measurement pattern temporarily persistent.

Figure 1 shows the average silhouette scores for k ranging between 2 and 8 was optimal for k=4, yielding an average silhouette score of 0.236, slightly higher compared to the silhouette score of k=3. Therefore, the cluster analysis yielded four distinct usage patterns based on usage over one year; we named these patterns “temporarily persistent (TP),” “stiff starting (SS),” “negligent quitting (NQ),” and “loyally persistent (LP).” Table 1 and Figures2^5^ provide an overview of the usage patterns.

**Table 1.** Details of the four usage patterns.

Usage pattern	Demographics (N=261)	Description	Adherence
TP^a^	62 (24): M^b^: 81% F^c^: 19%	Adherent start with all devices. Adherence decreases as time progresses, possible dropout.	47% (29/62) measured at least once in 80% (41.6/52) of the weeks. Dropout rate: 19% (12/62) after 6 months and 77% (48/62) after 12.
SS^d^	34 (13): M: 76% F: 24%	Nonadherent. After some weeks, adherent and the adherence declines, possible dropout.	68% (23/34) measured at least once a week for 80% (41.6/52) of the weeks. Dropout rate: 3% (1/34) after 6 months and 35% (12/34) after 12.
NQ^e^	97 (37): M: 70% F: 30%	Nonadherent start. Adherence decreases over time and often results in a dropout.	20% (19/97) measured at least once a week in 80% (41.6/52) of the weeks. Dropout rate: 20% (19/97) after 6 months and 66% (64/97) after 12.
LP^f^	68 (26): M: 85% F: 15%	Continuously adherent, with at least two devices. Maintained until the care track cessation.	100% (68/68) measured at least once in 80% (41.6/52) of the weeks. Dropout rate: 0% (0/68) after both 6 and 12 months.

a TP: temporarily persistent.

b M: male.

c F: female..

d SS: stiff starting

e NQ: negligent quitting.

f LP: loyally persisting.

**Figure 3.**
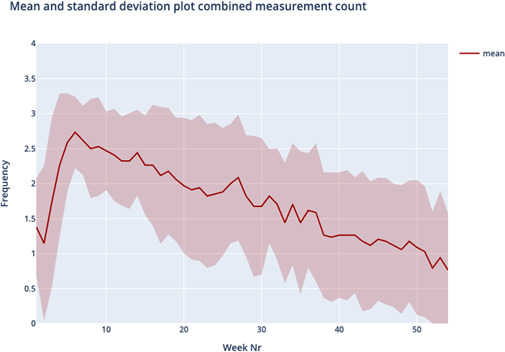
Measurement pattern stiff starting.

**Figure 4.**
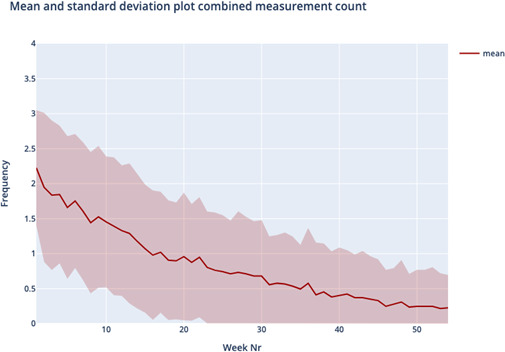
Measurement pattern negligent quitting.

**Figure 5.**
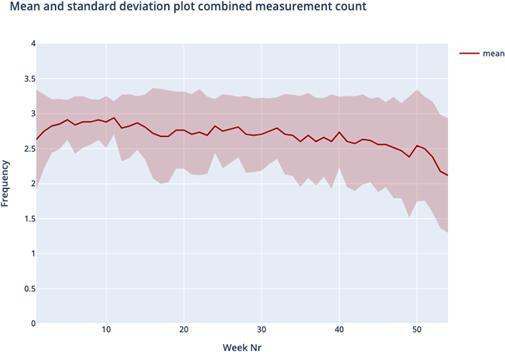
Measurement pattern loyally persisting.

### User Pattern Comparisons

The ANOVAs showed a significant difference in DBP between the clusters (NQ and LP and TP and NQ) in month 1 (F_3,245_=4.649, *P*=.004). The ANOVA also showed a significant difference in SBP between the clusters (NQ and LP and TP and NQ) in month 6 (*F*_3,199_=5.388, *P*=.001). Significance is determined at *P*<.013 (.05/4) after Bonferroni correction for multiple testing.

Additional contrast analysis, comparing the most adherent (LP) with the least adherent (NQ) user profile, showed that patients in the least adherent group had a significantly higher DBP in month 1 and 6 (DBP month 1: *T*_245_=-3.308, *P*=.001, *r*=0.21, DBP month 6: *T*_199_=−2.766, *P*=.007, *r*=0.19).

Additionally, a Kruskal-Wallis test was conducted, due to non-normal distribution, on the steps which yielded a significant difference in the number of steps in the first month among the clusters (*χ*^2^_3_=9.850, *P*=.020). A higher step count is seen in month one in TP and LP when compared to SS and NQ. The ANOVAs indicated no significant differences in weight between the clusters. Repeated measures ANOVAs did not show significant differences in changes in health outcomes in month 1‐6, 6‐12, and 1‐12 within each cluster. However, especially in month 12, there existed a substantial amount of missing data (see Table 2).

**Table 2.** Mean values of health outcomes per month, including the percentage available data in brackets.

CM and month		TP^a^ n (%)	SS^b^ n (%)	NQ^c^ n (%)	LP^d^ n (%)
Weight					
	1	84.9 (89)	86.8 (94)	87.6 (70)	84.7 (99)
	6	84.9 (65)	85.8 (79)	86.7 (35)	84.8 (99)
	12	91.2 (21)	86.3 (38)	91.8 (14)	83.4 (91)
Steps					
	1	4346 (98)^e^	3062 (85)^e^	3559 (66)^e^	4592 (97)^e^
	6	3883 (74)	4186 (59)	4331 (23)	4861 (97)
	12	4028 (24)	5339 (29)	3885 (5)	4911 (93)
SBP^f^					
	1	124.5 (98)	127.2 (100)	127.2 (89)	124.1 (100)
	6	122.9 (74)^e^	126.5 (97)	130.7 (58)^e^	123.5 (100)^e^
	12	125.4 (31)	125.0 (71)	128.7 (29)	123.1 (97)
DBP^g^					
	1	74.5 (98)^e^	75.6 (100)	78.5 (89)^e^	74.1 (100)^e^
	6	74.1 (74)	75.9 (97)	78.7 (58)	74.3 (100)
	12	77.3 (31)	74.5 (71)	77.6 (29)	74.2 (97)

a TP: temporarily persistent.

b SS: stiff starting.

c NQ: negligent quitting.

d LP: loyally persisting.

e A significant difference between the clusters.

f SBP: systolic blood pressure.

g DBP: diastolic blood pressure.

### Generative Interviews

#### Demographics

In total, 18 patients were recruited, two canceled their participation, resulting in 16 patients being interviewed. Out of 16, 14 (87.5%) were men and the average age was 60.6 (SD 9.7). Of these patients, 8 (50%) were halfway through the care track and 8 (50%) were approaching the end of the care track. Based on their usage patterns, 6 patients were classified as TP, 2 as SS, 2 as NQ, and 6 as LP (For demographics of interviewed participants, see Multimedia Appendix 2).

#### Adherence Influencing Factors

**Figure 6.**
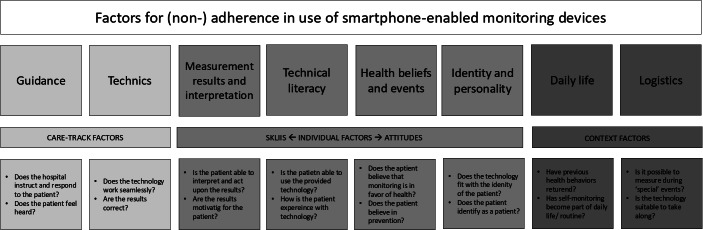
Factors for (non)adherence derived from the interview.

Qualitative analysis in Atlas.ti resulted in 536 codes. Thematic analysis of the codes resulted in eight reasons for adherence, combined into three general factors: care track (guidance and techniques), individual (measurement results and interpretation, technological literacy, health beliefs and events, and identity and personality), and context factors (daily life and logistics). The questions below the factors are to further explain the meaning of each factor and possible questions to uncover these factors within the patient population (Figure 6).

#### Care Track Factors

##### Guidance

Clear instructions about and response to measurements from the hospital was an important factor for adherence. 11 patients expressed positive experiences with guidance from the hospital: “It is very nice that you are connected to the hospital, they directly called me back the other day!” (TP). In contrast, 5 patients indicated guidance was lacking: “They did not give me any instructions; I just had to see for myself” (SS).

##### Technics

Well-functioning devices lower the threshold for self-management, whereas malfunctioning often resulted in non-adherence. For 9 patients, the devices and app worked seamlessly: “We already had a scale at home, but the provided scale registers everything very nicely” (LP). However, seven patients faced technical problems: “When the devices do not work and are not connecting, I do not know what to do anymore. I have been trying enough for now” (TP).

### Individual Factors

#### Measurement Results and Interpretation

When patients can interpret the results and consider them meaningful, adherence is more likely. For 12 patients, the outcomes and their interpretations of the measurements were a motivator for measuring: “It is very calming that you see that your blood pressure is stable and your heart rate still good” (LP). In contrast, the measurement results were demotivating for four patients: “If everything was in the red, I thought to myself wat can I do? Should I call the doctor? What can I do?” (TP).

#### Technological Literacy

The degree of technological literacy and available support can play a critical role in adherence. During the interviews, 11 patients expressed having sufficient experience with technology: “As a physicist, you can call me a ‘techie’” (LP). Patients would often receive support from their partner or family members, which made using the devices still doable for them. Five patients indicated a lack of technological literacy: “I never use the app; I am just not able to do that” (NQ).

#### Health Beliefs and Events

For patients to be intrinsically motivated, they should consider the intervention as relevant for their own health and avoiding future cardiovascular or health events. 11 patients indicated that their infarction and health was a reason for measuring: “When I have health complaints, I just do some extra checks with the devices” (SS). However, five patients did not see the value of measuring for their own health currently and believed that avoiding future events was not greatly influenceable by their own health behavior: “Well, the kilo’s do not really matter to me and my health” (TP).

#### Identity and Personality

It is important that the eHealth intervention aligns with the patient’s identity for adherent use. Six patients indicated that the devices matched with their personal identity and identity as a patient. “I have just had an infarction, so it is a fact that I am a patient. I accepted that; it is part of my life now” (LP). On the other hand, 4 patients rejected a patient identity: “I also should not be always labeling myself as a heart patient (SS), or that the smartwatch did not fit their personal style: “For my job, I need to dress appropriately, and the pedometer watch did not fit my style” (SS).

### Context Factors

#### Daily Life

Whether using the intervention has become part of daily practice can influence adherence. This was the case for 12 patients: “I always measure around 9AM when I just had my breakfast, then I do all the measurements. I also took my medication at that time” (LP). For 4 patients, measuring did not become a habit or was forgotten: “The last weeks I did not do all the measurements, I had a lot of stress at my work. Then I just cannot do it” (LP).

#### Logistics

It is essential that the telemonitoring devices are easy to handle in varying contexts to stimulate adherent use. Seven patients expressed themselves positively regarding logistics. They valued that The MI Box reduced their travel time to the hospital and that the devices were easy to take with them: “I do not like to travel a lot, as I always face many obstacles such as tractors, which makes me nervous. Therefore, I really like that I can just do the measurements at a distance” (LP). However, three patients indicated logistical issues which led them not to do their prescribed measurements: “Well, then you have to measure three times when you are on a holiday, I am never going to do that” (LP).

When placing the factors listed in Figure 6 alongside the usage patterns, trends can be observed. Participants in the LP group mentioned that for the majority, all factors contributing to their adherence. Participants in the TP group showed a more divided perspective on factors for adherence. Most participants found guidance, measurement results and interpretation, daily life, and logistics a factor for their adherence. This group is divided on technological literacy and identity and personality. Participants in the NQ group, guidance was a factor for adherence. However, many mentioned health beliefs and measurement results and interpretations to be factors for their nonadherence. Finally, participants in the SS group identified several factors for adherence including technical issues, measurement results and interpretation, technological literacy, health belief, and events. However, guidance was mentioned as the factor for nonadherence. The distribution per usage pattern of the number of participants who mentioned the factor as a reason for adherence and in parentheses those who mentioned it as a factor for nonadherence can be seen in Table 3.

**Table 3.** Number of participants per usage pattern factor for adherence (and nonadherence in parentheses).

Factor	LP^a^ (N=6)	TP^b^ (N=6)	NQ^c^ (N=2)	SS^d^ (N=2)
Guidance	5 (1)	4 (2)	2 (0)	0 (2)
Technical Issues	5 (1)	1 (5)	1 (1)	2 (0)
Measurement results and interpretation	6 (0)	4 (2)	0 (2)	2 (0)
Technological literacy	5 (1)	3 (3)	1 (1)	2 (0)
Health beliefs and events	6 (0)	4 (2)	0 (2)	2 (0)
Identity and personality	2 (1)	2 (1)	0 (1)	1 (1)
Daily life	6 (0)	4 (2)	1 (1)	1 (1)
Logistics	2(1)	5 (1)	0 (1)	1 (0)

a LP: loyally persisting.

b TP: temporarily persistent.

c NQ: negligent quitting.

d SS: stiff starting.

### Persona Development

Four personas were developed based on the results from the database analysis and generative interviews, named Tamara, Peter, Sam, and Kim. These personas are composed of usage patterns enriched by interview data to provide a rich description of the patient population. Furthermore, each persona contains selected factors from the interviews that are either important or unique within this usage pattern. These personas are not descriptive of all within each usage pattern and are not an identical copy of the data; however, they provide an impression of patients and their concerns with the eHealth intervention (see Multimedia Appendix 3).

#### Tamara

Tamara represents the loyally persisting usage pattern. The SHMDs motivate her to get and stay healthy. The two factors essential to Tamara are patient identity and routine. Namely, Tamara is okay with being a patient and has built eHealth into her daily routine. Tamara can run the risk of overtesting to keep a sense of control and reduce her feelings of panic about her health. The change in Tamara’s health outcomes (which represents the average LP user) is shown in Table 4.

**Table 4.** Average change in health outcomes per persona.

Persona and clinical measure		Month 1	Month 6	Month 12
Tamara				
	Weight (kg)	85	85	83
	Steps	4592	4861	4911
	SBP^a^ (mm Hg)	124	124	123
	DBP^b^ (mm Hg)	74	74	74
Peter				
	Weight (kg)	85	85	91
	Steps	4346	3883	4028
	SBP (mm Hg)	125	123	125
	DBP (mm Hg)	75	75	77
Sam				
	Weight (kg)	85	85	91
	Steps	4346	3883	4028
	SBP (mm Hg)	125	123	125
	DBP (mm Hg)	75	75	77
Kim				
	Weight (kg)	87	86	86
	Steps	3062	4186	5339
	SBP (mm Hg)	127	127	125
	DBP (mm Hg)	76	76	75

a SBP: systolic blood pressure.

b DPB: diastolic blood pressure.

#### Peter

Peter represents the temporarily persisting usage pattern. For Peter, the intervention must be easy, and he does not experience the SHMDs to work seamlessly. The 2 factors essential to Peter are support and technological literacy. Peter has a strong support network of friends and family. However, he is unsure about his technological skills. The change in Peter’s health outcomes (average TP user) is shown in Table 4.

#### Sam

Sam represents the negligently quitting usage pattern. Sam does not see the use of the SHMDs, especially when he is not experiencing any symptoms. The two factors essential to Sam are identity and routine. Sam does not feel like a patient and often forgets to measure. The change in Sam’s health outcomes (average NQ user) is shown in Table 4.

#### Kim

Kim represents the stiff starting usage pattern. At first, Kim did not know all the functions and features of the devices and found it hard to figure out. The two important factors for Kim are technological literacy and support. Kim has confidence in her technological skills, but she does not feel supported by the hospital. The change in Kim’s health outcomes (average SS user) is shown in Table 4.

## Discussion

### Principal Findings

The aim of this study was to identify user patterns to improve adherence to RPM interventions in patients after MI. The identified 4 distinct usage patterns are consistent with the literature which found similar usage patterns based on usage data of interventions within different populations, differentiating between those users who do interact, who sparingly interact, and highly interact with technology [15,16]. These usage patterns can help healthcare professionals to identify those patients who are less reached by technology and to identify strategies to improve their adherence to RPM interventions after MI.

Analysis of health outcomes based on these usage pattern clusters showed a significant difference in BP between the patterns (NQ and LP and LP and TP) in month 1 and 6 and for steps in month one (LP and TP and SS and NQ), with a trend of lower blood pressure and higher step count in the high adherence groups. This shows the potential for tailoring to improve health outcomes throughout the whole care track and between different patterns. The differences between usage patterns in months 1 and 6 are possible moments of interest for tailoring to ensure an advantageous start and continuation. Furthermore, around month 6, patients conclude rehabilitation; therefore, this may be an important moment to address adherence and motivation of users to continue with the intervention. When comparing the most to the least adherent usage patterns, a significantly higher DBP was seen in the least adherent pattern. The observed relation between adherence and better-controlled BP can be explained in several ways. First, medication intake might correlate with device usage [43]. Second, monitoring BP can lead to an increase in healthy lifestyle [44]. Finally, repeated high BP values might demotivate adherently performing the required measurements, as the measurements’ results are unsatisfactory [18]. Thus, those with less controlled BP may become less adherent over time. When observing the health outcomes within month one, a possible trend can be seen, namely that users with a “good start” tend to have “good results.”

The interviews resulted in care track, individual, and context factors of nonadherence. These findings are consistent with literature [18]. Previous findings have similar aspects for nonadherence, including frustrating technology, perception of content, and support through face-to-face contact [45]. Furthermore, a previous study found similar factors as values of importance for this population and are recommendations for further eHealth development for this population [46].

These findings led to the creation of four personas named Tamara, Kim, Peter, and Sam. These personas are specific to this patient population. However, previous literature within different populations uses similar mixed methods approaches [27,29,47]. Based on these findings, we formulate the following tailoring recommendations. These recommendations were specified per persona; however, these can apply across the care track and for other eHealth interventions. Further research can be conducted on the efficacy of these tailoring measures.

For users such as Peter, the essential factor for adherence is support, and for nonadherence, it is technological literacy. Therefore, comprehensive technology and engagement strategies can be used to tailor the intervention. The more confident a user is in their ability, the more likely they will perform well within the intervention [48]. Furthermore, self-efficacy influences eHealth use and is valued by patients with cardiovascular disease [46,48,49]. Although avoiding all technological difficulties and frustrations is not possible, it is important that users feel that they can reach out for guidance. Therefore, a tailored measure can be to emphasize to these users that technological issues may arise and that this has nothing to do with their competencies; moreover, reaching out to is encouraged. Staff could monitor usage in the first week and be on standby to provide support when nonadherence is observed. Furthermore, these users can be given more time when starting the care track to ask questions and test-run the devices. For users like Kim, whose essential factor for non-adherence is desire for more guidance and support, a recommendation is to provide face-to-face interaction with the health care provider [50]. However, this could be difficult given time constraints of health care providers. Therefore, a recommendation can be to provide the user with a “buddy” or a support group of other participants [51] to which they feel accountable. This buddy can also come directly from the user’s social environment [46]. This tailoring measure is essential at the start of the intervention since these users typically start in a non-adherent state. Users like Sam, whose essential factor for nonadherence is identity and routine, can be harder to impact through alterations in the design of the intervention. However, prior to starting the intervention, steps can be made to change their perception and motivation. Tailored, inclusive health education by trusted healthcare professionals or through testimonials from previous users can potentially increase health literacy and their perception of the importance of the intervention [24]. A study on eHealth interventions for smoking cessation showed that those less motivated also engaged less with devices and smartphone applications. Indicating that, regardless of the design elements of the intervention, motivation partially determines engagement. This study indicated that although lack of motivation significantly reduces the engagement of the participant, it is not reduced to zero; therefore, it is critical to find alternative “low-effort communication” [52]. Although relevant to all participants, empowerment strategies could potentially increase these users’ adherence. These strategies can include but are not limited to goal setting, feedback on behavior, information about health consequences, social support, and demonstrations of the behavior [48]. Loyal users such as Tamara, whose essential factor for adherence is identity and routine, can provide essential information for improvements to make eHealth more user-friendly. For example, within the interviews, a participant indicated that they forget to do measurements when there is stress in their daily lives. Therefore, providing information on how to continue with measurements when stress is high or how to handle stress could lead to even more consistent use. Furthermore, these users can be supported by further enhancing her autonomy by giving more decision room in the care track [46]. These personas also highlight the potential significance effects of culture and contextual factors on RPM experience and effectiveness; therefore, future research should explore these effects specifically. Focusing on the factors that are crucial to increase adherence and are desirable for a personalized experience.

The study design allowed the obtainment of the aim, to develop personas of patients using SHMDs. Using quantitative insights as the foundation enriched with qualitative insights allowed an in-depth understanding of how SHMDs are used and experienced. The identified usage patterns, adherence factors, and personas are based on objective self-management data and subjective user experience data and thereby provide a deeper understanding of the users and the potential for tailoring. The study approach built upon a by a study of Ten Klooster et al [17], which indicated that meaningful usage patterns can be created through using quantitative data and qualitative insights. Although the study provides a clear stratification and deeper insights, there are limitations worth mentioning. First, the identification of clusters was based on the presence or absence of measurements. Consequently, for nonadherent patients, much data was missing. Therefore, the exploration of CMs was done on the means of month 1, 6, and 12. Second, since no measures were undertaken to predict cluster membership or to apply the results to new data, one should be cautious in generalizing the results beyond the sample. Third, the distribution of user patterns within the interview sample is not equal; however, this was taken into consideration when creating the personas to include narrative aspects from all patterns. In addition, the interview part of this study involved a relatively small sample size; however, due to the in-depth nature of the interview, we were able to get rich insights into patient experience on adherence. Finally, the male sex made up most of the participants within the study, specifically, the interview study included only 2 females. This could have influenced the creation of the personas, since it has been shown that males and females interact differently with the health care system and providers [53-55].

### Conclusion

The goal of this study was to unlock the personalizing potential of RPM eHealth interventions in motivating and personally meaningful care for patients with MI. The identified usage patterns can help health care professionals to identify those patients who are less reached by technology and to identify strategies to improve their adherence to RPM interventions after MI. This study identified 4 usage patterns and personas, namely temporarily persisting Peter, stiff starting Kim, negligently quitting Sam and loyally persisting with Tamara, provides insights into their reasons for adherence. These personas can assist healthcare professionals in tailoring interventions to the patient subpopulations, aiming at higher adherence and effectiveness of interventions. The usage patterns can indicate whether a patient may drop out, which can result in losing overview of this patient or, as this study suggests, a worsening of BP control. The study provides a deeper understanding of the heterogenous patient population and, to our knowledge, is the first to publish usage patterns and personas for this type of eHealth intervention. Next steps will include using these personas to tailor the RPM intervention to the individual with the aim to improve overall adherence and clinical outcomes.

## Supplementary material

10.2196/56236 Multimedia Appendix 1 Decision tree.

10.2196/56236 Multimedia Appendix 2 Demographics of interviewed participants.

10.2196/56236 Multimedia Appendix 3 The box personas.

## ABBREVIATIONS

BP: blood pressure
CM: clinical measures
CVD: cardiovascular disease
DBP: diastolic blood pressure
DTW: dynamic time warping
ECG: electrocardiography
EMR: electronic medical record
GDMT: guideline-driven medical therapy
LP: loyally persisting
LUMC: Leiden University Medical Center
MI: myocardial infarction
NQ: negligent quitting
NST-ACS: non-ST acute coronary syndrome
RPM: remote patient management
SBP: systolic blood pressure
SHMD: smartphone-enabled health monitoring devices
SS: stiff starting
STEMI: ST-elevation myocardial infarction
TP: temporarily persisting
