# Supplementary figures and images for: Adherence Patterns of Patients Using Remote Patient Management After Myocardial Infarction: Mixed Methods Persona Approach
Source: JMIR Cardio. 2025 Aug 18;9:e56236. doi: 10.2196/56236 (PMC12360670; doi:10.2196/56236)

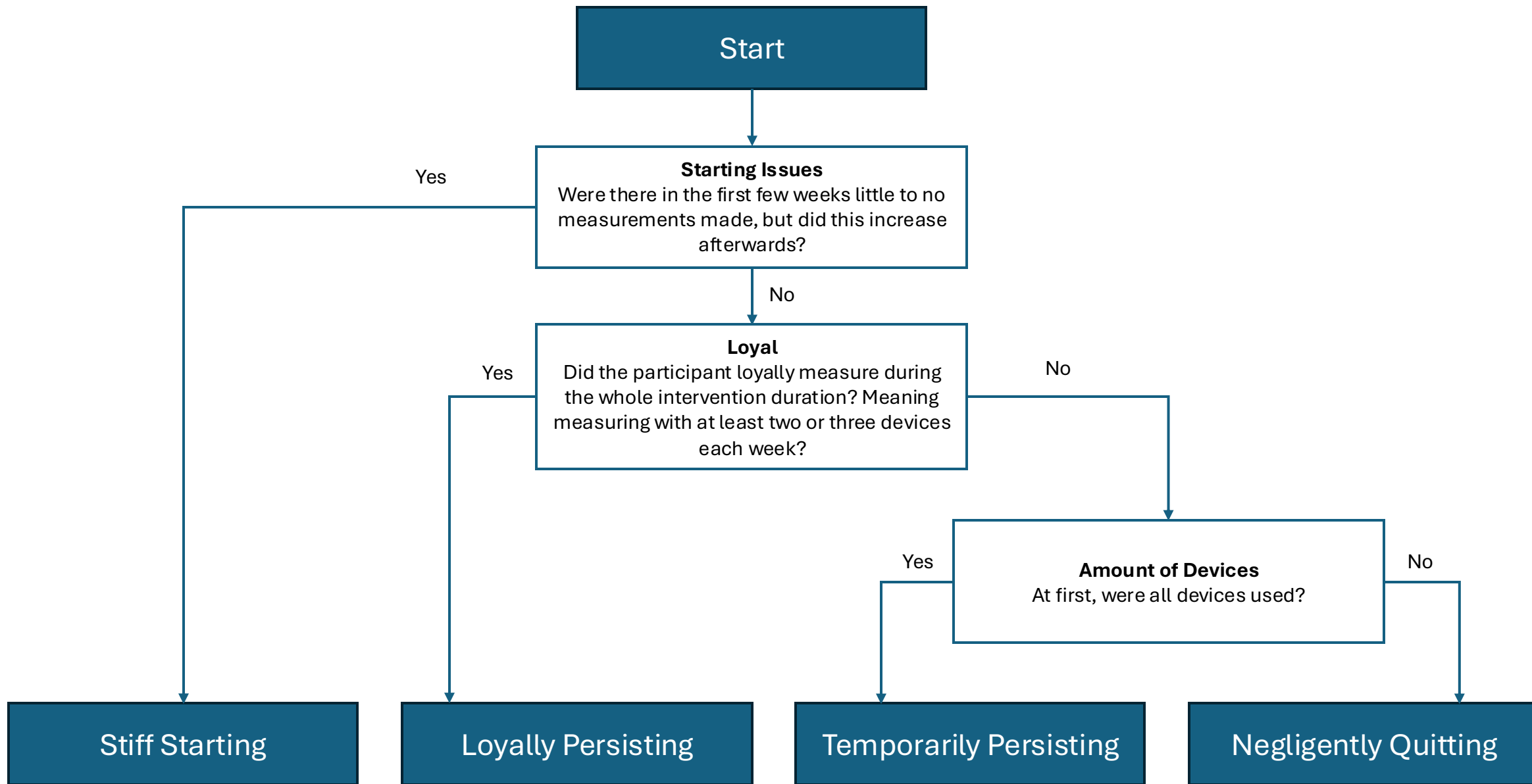

Supplement: Multimedia Appendix 1 [file cardio-v9-e56236-s001.pdf]
