# Supplementary material for: Adherence Patterns of Patients Using Remote Patient Management After Myocardial Infarction: Mixed Methods Persona Approach
Source: JMIR Cardio. 2025 Aug 18;9:e56236. doi: 10.2196/56236 (PMC12360670; doi:10.2196/56236)
Supplement: Multimedia Appendix 2 [file cardio-v9-e56236-s002.docx]

Appendix One: Demographics of interviewed participants.

| Participant | Gender | Age (years) | Follow-up (months) | User-Profile |
| --- | --- | --- | --- | --- |
| 1 | M | 47 | 6 | LP |
| 2 | M | 55 | 6 | SS |
| 3 | M | 72 | 12 | NQ |
| 4 | F | 74 | 12 | TP |
| 5 | M | 65 | 6 | LP |
| 6 | M | 58 | 12 | SS |
| 7 | M | 56 | 12 | TP |
| 8 | M | 51 | 6 | LP |
| 9 | M | 43 | 12 | NQ |
| 11 | F | 66 | 6 | TP |
| 12 | M | 56 | 12 | TP |
| 13 | M | 74 | 12 | TP |
| 15 | M | 50 | 6 | TP |
| 16 | M | 72 | 6 | LP |
| 17 | M | 64 | 6 | LP |
| 18 | M | 67 | 12 | LP |

#### key:

*TP: temporarily persistent*

*SS: stiff starting*

*NQ: negligent quitting*

*LP: loyally persisting*

*M: male*

*F: female*
