# Supplementary material for: Adherence Patterns of Patients Using Remote Patient Management After Myocardial Infarction: Mixed Methods Persona Approach
Source: JMIR Cardio. 2025 Aug 18;9:e56236. doi: 10.2196/56236 (PMC12360670; doi:10.2196/56236)
Supplement: Multimedia Appendix 3 [file cardio-v9-e56236-s003.pdf]

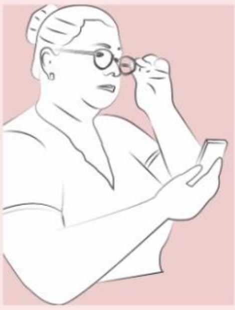

Tamara

"The Box motivates me to get and stay healthy"

I'm 65 and living with my husband in Leiden. We have two daughters & one grandchild.

I really enjoy using The Box. Measuring every week has given me a feeling of security. I quickly accepted that I was a heart patient and want to do everything in my power to improve my health.

I have a set time when I measure. Sometimes when I am stressed or have to go out of the house, I don't measure at the set time; however, I always catch up at a later moment. I plan to use The Box for the full year and even after to keep an eye on my health.

Tamara's adherence pattern

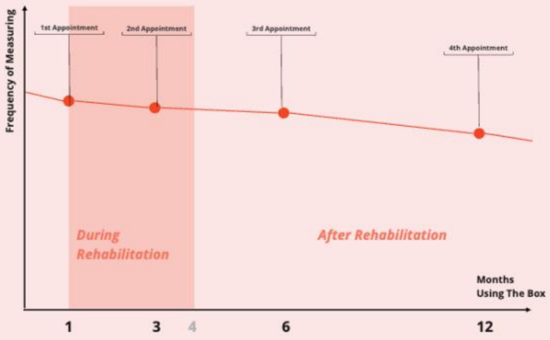

Key factors

I have a routine

I am a heart patient

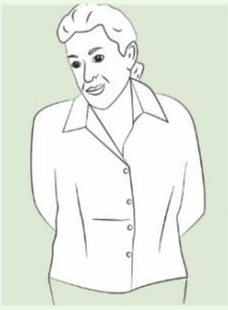

Kim

"I did not know what to do with the Box at first"

I am 62 years old and live in Leiden. I have three children with my ex-husband.

When I was in the hospital, the staff was amazing and very supportive. After I was discharged, I felt overwhelmed about all the things I needed to do. It took me a while to figure out how to use The Box at home, but eventually I figured it out. The care team at the rehabilitation center were a great support.

However, as I left rehabilitation and time went on, I felt less and less supported by LUMC. I send my measurements every week but did not get a lot of feedback. After a while, I started measuring every so often when I remembered.

Kim's adherence pattern

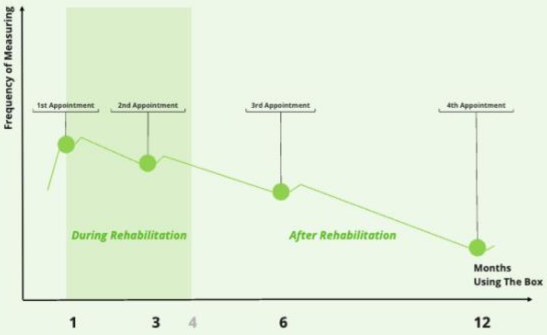

Key factors

I am confident with technology

I want to feel supported by LUMC

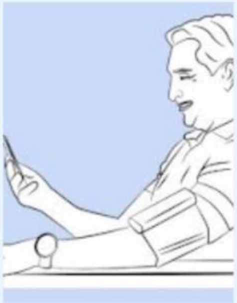

Peter

"I like the Box, but it's not always easy"

I am 58 years old, and live in Leiden with my wife and dog.

I am not very technologically savvy, but my wife helps me with the measurements.

I really like seeing my measurements. It helps me feel more in control about my health. However, I sometimes have problems connecting to the LUMC care application. My wife also doesn't know how to fix it. I can sometimes get so frustrated by the technology that I will stop using The Box for a while.

Peter's adherence pattern

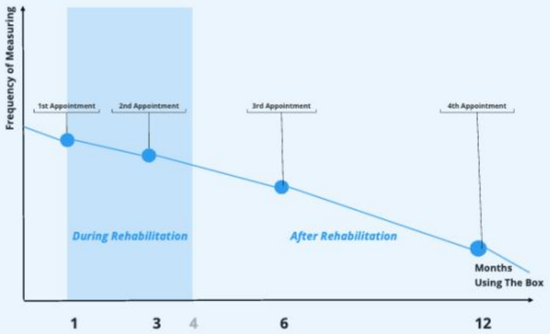

Key factors

I am not confident with technology

My family & friends are great support

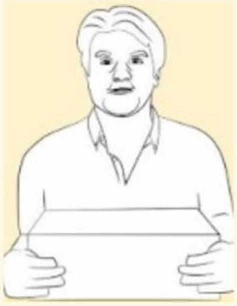

Sam

"I don't have symptoms, so I don't use the Box."

I am 70 years old, and I live with my wife in Leiden.

When I received The Box, I found it quite interesting and flashy. I started measuring with the different devices after I got out of the hospital.

However, now I feel fine and don't really have symptoms, so I do not see the use in measuring. I want to move forward with my life.

Sam's adherence pattern

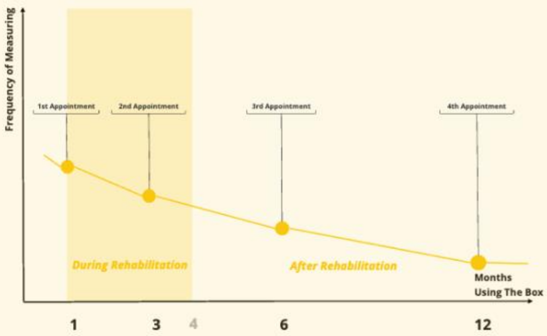

Key factors

I don't feel sick anymore, so I do not need to measure

I don't want to feel like a patient
